# Small molecule activation of NOTCH signaling inhibits acute myeloid leukemia

**DOI:** 10.1038/srep26510

**Published:** 2016-05-23

**Authors:** Qi Ye, Jue Jiang, Guanqun Zhan, Wanyao Yan, Liang Huang, Yufeng Hu, Hexiu Su, Qingyi Tong, Ming Yue, Hua Li, Guangmin Yao, Yonghui Zhang, Hudan Liu

**Affiliations:** 1 Hubei Key Laboratory of Natural Medicinal Chemistry and Resource Evaluation, School of Pharmacy, Tongji Medical College, Huazhong University of Science and Technology, Wuhan, 430030, China; 2 Department of Pharmacy, Wuhan Children’s Hospital, Wuhan, 430016, China; 3 Department of Hematology, Tongji Hospital, Wuhan, 430030, China; 4 School of Basic Medicine, Tongji Medical College, Huazhong University of Science and Technology, Wuhan, 430030, China; 5 Medical Research Institute, Wuhan University, Wuhan, 430071, China

## Abstract

Aberrant activation of the NOTCH signaling pathway is crucial for the onset and progression of T cell leukemia. Yet recent studies also suggest a tumor suppressive role of NOTCH signaling in acute myeloid leukemia (AML) and reactivation of this pathway offers an attractive opportunity for anti-AML therapies. *N*-methylhemeanthidine chloride (NMHC) is a novel Amaryllidaceae alkaloid that we previously isolated from *Zephyranthes candida*, exhibiting inhibitory activities in a variety of cancer cells, particularly those from AML. Here, we report NMHC not only selectively inhibits AML cell proliferation *in vitro* but also hampers tumor development in a human AML xenograft model. Genome-wide gene expression profiling reveals that NMHC activates the NOTCH signaling. Combination of NMHC and recombinant human NOTCH ligand DLL4 achieves a remarkable synergistic effect on NOTCH activation. Moreover, pre-inhibition of NOTCH by overexpression of dominant negative MAML alleviates NMHC-mediated cytotoxicity in AML. Further mechanistic analysis using structure-based molecular modeling as well as biochemical assays demonstrates that NMHC docks in the hydrophobic cavity within the NOTCH1 negative regulatory region (NRR), thus promoting NOTCH1 proteolytic cleavage. Our findings thus establish NMHC as a potential NOTCH agonist that holds great promises for future development as a novel agent beneficial to patients with AML.

Acute Myeloid Leukemia (AML), the most common type of acute leukemia diagnosed in adults and second most common in children, is a highly aggressive hematological malignancy that originates from hematopoietic stem cells and myeloid progenitors^1^. Conventional chemotherapies, including cytosine arabinoside (Ara-c) or combination with other reagents, often become ineffective due to the heterogeneity of leukemia cells^2^. Significant advances have been made to understand the molecular pathogenesis and brought a new perspective for targeted therapies, such as clinical application of FLT3 inhibitors^3^, yet relapse remains the most common reason for treatment failures^4^. Remaining a daunting threat, AML requires an urgent exploration for novel therapeutic strategies. Recent studies reported activation of the NOTCH pathway, mainly through administration of peptides mimicking NOTCH ligands, would inhibit AML cell propagation and survival^5^,^6^. NOTCH is a transmembrane receptor, activation of which is normally initiated by interaction with a membrane ligand from a neighbor cell. This association elicits proteolytic cleavages, terminating in γ-secretase-mediated generation of intracellular NOTCH (ICN) that activates responder gene expression in the nucleus^7^. It is believed that activation of NOTCH1 in AML induces the expression of downstream gene *HES1*, which transcriptionally represses major oncogenes such as *FLT3*^8^. These findings strongly suggest that therapeutic targeting of NOTCH could be a potentially effective approach to combat master oncogenic drivers in AML thus likely beneficial for leukemia patients.

Herbaceous plants have been widely used as therapeutic agents for disease prevention and treatments. Plants of the family Amaryllidaceaes have long been recognized for their medicinal values^9^ and *Zephyranthes candida* (Lindl.) Herb. is an amaryllidaceous bulbous herb and used to treat infantile convulsions, epilepsy, and tetanus^10^,^11^,^12^. We previously reported the anti-tumor activity of Amaryllidaceae alkaloids isolated from the whole plant of *Z. candida*^10^ and found that *N*-methylhemeanthidine chloride (NMHC) exhibited cytotoxicity to multiple cancer cell lines derived from a variety of tumor origins^10^,^13^, especially the AML cell line HL-60.

In this study, we have analyzed multiple leukemic cells and found that cell growth rates of acute myeloid leukemia, but not lymphoid leukemia or chronic myeloid leukemia, are markedly inhibited by NMHC, which induced both cell cycle arrest and apoptotic death. To explore the molecular basis of NMHC-mediated anti-AML effect, we systematically analyzed differential gene expression between DMSO and NMHC-treated AML cells. Genome-wide gene expression profiling revealed that genes involved in the NOTCH signaling pathways are up-regulated by NMHC. Combined biochemical and molecular modeling studies demonstrated NMHC bound to the negative regulatory region (NRR) of NOTCH1, facilitating protease-mediated NOTCH processing and ICN production. These findings thereby identify NMHC as a potential chemical compound capable of activating NOTCH, which may be used to substitute the current peptide-based NOTCH activation and be considered further evaluation of its anti-leukemic role with a hope to benefit AML patients.

## Results

### NMHC specifically inhibits AML cell proliferation

**Figure 1:**
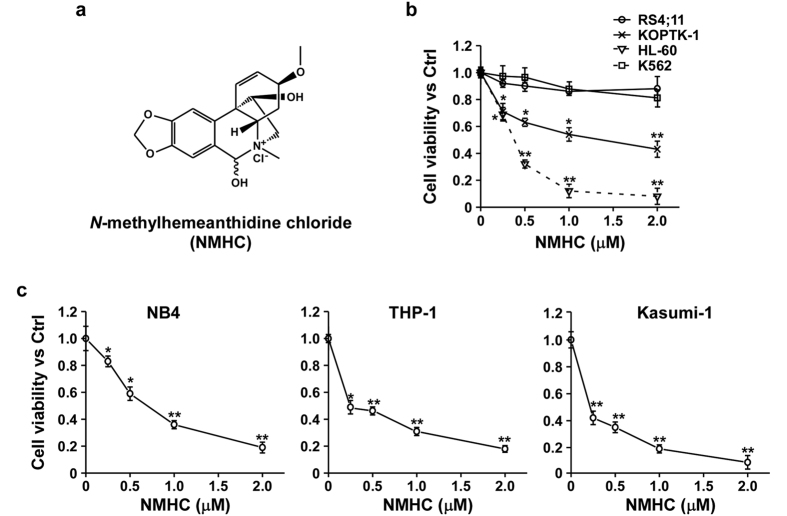
*N*-methylhemeanthidine chloride (NMHC) suppressed acute myeloid leukemia cell proliferation. (**a**) The chemical structure of NMHC. (**b**) Effects of NMHC on viability of a variety of hematological malignant cells. (**c**) Effects of NMHC on acute myeloid leukemia cell viability. Above all, designated cells were treated with indicated concentrations of NMHC for 48 h and cell viability was evaluated using CCK8 kits. Relative cell viability is presented as an average ratio to PBS treatment from triplicate wells ± SD. *P*-values were derived from Student’s *t*-test (*P < 0.05, **P < 0.01; NMHC treatments vs DMSO treatments).

Small molecule compound NMHC used in our cellular assays has been purified from *Z. candida* with structure determined by NMR^10^ (Fig. 1a) and purity (99.1915%) assessed by HPLC^13^. Our prior studies suggest anti-tumor activity of NMHC in a variety of cancer cells, among which leukemia cell line HL-60 is most sensitive to NMHC-mediated cytotoxicity^10^. We then surmised that NMHC was specifically cytotoxic to hematological malignancies. To test this, we evaluated cell viability of a variety of leukemia cells, including acute B-cell lymphoblastic leukemia (B-ALL) cell RS4;11, acute T-cell lymphoblastic leukemia (T-ALL) cell KOPTK1, chronic myeloid leukemia (CML) cell K562 and acute myeloid leukemia (AML) cell HL-60, upon NMHC treatments. As shown in Fig. 1b, increasing concentrations of NMHC led to marked growth inhibition of HL-60 while exhibited only moderate effect on KOPTK1 and minimal influence on RS4;11 as well as K562. We further tested three additional AML cell lines NB4, THP-1 and Kasumi-1 in the presence of NMHC. Consistently, NMHC caused a substantial growth inhibition in all AML cell lines tested (Fig. 1c). Taken together, these data manifest that NMHC has a specific and strong cytotoxicity to AML cells.

### Administration of NMHC induces AML cell cycle arrest and apoptotic death

**Figure 2:**
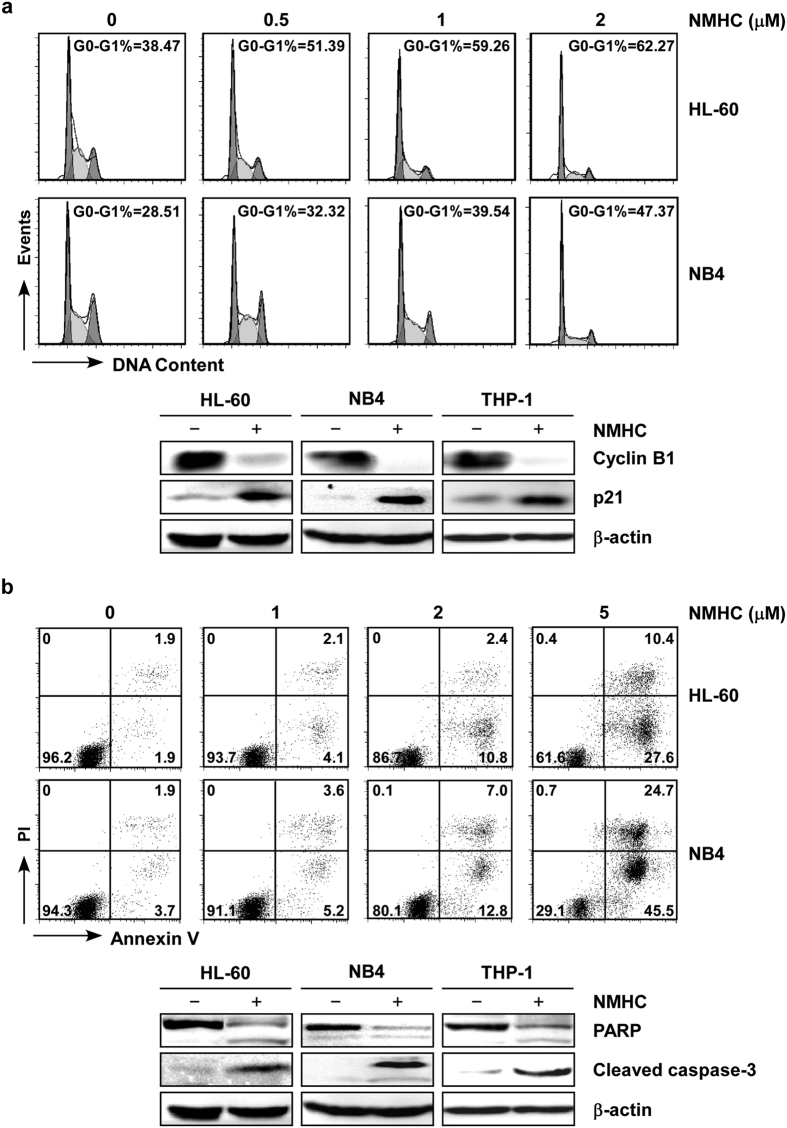
NMHC induced cell cycle arrest and cell apoptotic death in AML. (**a**) Analysis of cell cycle distributions in NMHC-treated cells. Cells were treated with various doses of NMHC for 24 h, followed by cell cycle analysis. The percentages of G0-G1 phases are shown in each graph. Effect of NMHC on cell cycle-relevant proteins was shown in the bottom. Cyclin B1 and p21 were detected by immunoblotting after 24 h treatments. (**b**) Analysis of apoptotic cell death. Cells were treated with various doses of NMHC for 24 h, stained with Annexin V-FITC/PI and analyzed by flow cytometry. Apoptosis-relevant proteins PARP and cleaved Caspase-3 were assessed by immunoblotting after DMSO or NMHC (1 μM) treatments for 24 h. Above all, representative data from three independent experiments are shown. β-actin is detected as the internal reference.

To explore the mechanisms of NMHC-induced growth inhibition, we examined the cell cycle distributions of HL-60 and NB4 cells treated with increasing doses of NMHC for 24 h. Administration of NMHC caused a substantial expansion of G0/G1 phase with a concomitant decrease of S and G2 distributions (Fig. 2a), manifesting a blockade of cell cycle at the G0/G1 phase. Further examination of cell cycle relevant proteins revealed decreased Cyclin B1 and increased cyclin-dependent kinase inhibitor 1 (p21) expression in HL-60, NB4 and THP-1 cells (Fig. 2a), confirming cell cycle arrests at the G1 phase. We next assessed apoptotic cell death induced by NMHC. As shown in Fig. 2b, the percentage of Annexin V positive populations, representing cells undergoing apoptosis, drastically went up in an NMHC dose-dependent manner. Consistently, augmented gene expressions of PARP and cleaved Caspase-3 were observed upon NMHC treatments (Fig. 2b). These results demonstrate that NMHC not only impedes cell division but also induces apoptotic cell death in AML cells.

### NMHC affects gene expression involved in AML, apoptosis and the NOTCH signaling pathway

**Figure 3:**
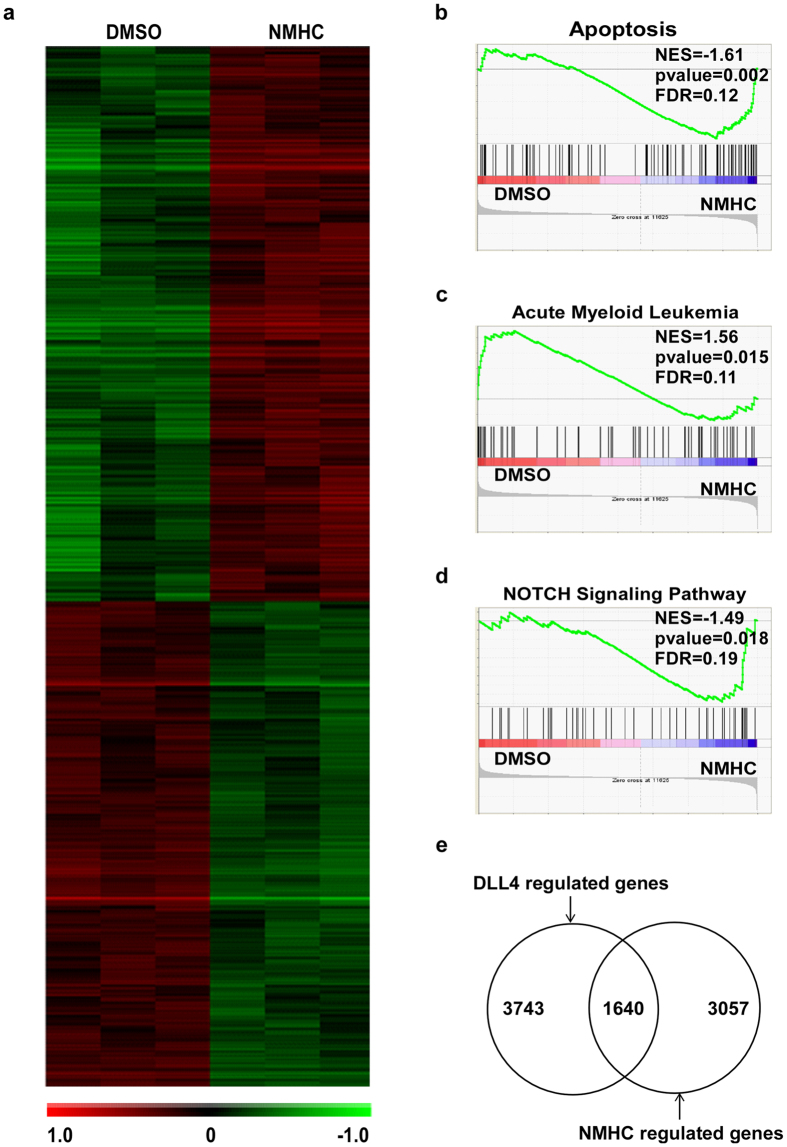
NMHC treatments affected expression of genes involved in apoptosis, acute myeloid leukemogenesis and the NOTCH signaling pathway. (**a**) Heatmap presentation of altered gene expression upon NMHC treatments. Addition of 1 μM NMHC in HL-60 cell culture was followed by total RNA preparation and microarray analysis. Differential gene expression was clustered as shown. GSEA analysis of apoptosis (**b**), acute myeloid leukemia (**c**) and the NOTCH signaling pathway (**d**) was presented. (**e**) Comparison of NMHC-modulated genes and DLL4-regulated genes derived from GSE42261^6^.

To gain mechanistic insights into NMHC-mediated anti-AML activity, we compared whole transcriptome profiling between DMSO or NMHC-treated HL-60 cells (See materials and methods). 4697 differentially expressed genes were selected using the criteria of fold changes (>1.2 or <−1.2) and *p*-values (*p* < 0.05) (Fig. 3a). Among those genes, we found apoptosis-relevant genes were significantly enriched upon administration of NMHC (Fig. 3b), consistent with the observation shown in Fig. 2b. Gene set enrichment analysis (GSEA) also revealed that gene signatures associated with AML development were significantly down-regulated by NMHC (Fig. 3c). Deep analysis of the gene expression profile revealed an up-regulated enrichment of gene signatures involved in the NOTCH signaling pathway (Fig. 3d), reminiscent of recent reports suggesting tumor suppressive activity of NOTCH in AML^5^,^6^. To confirm the specificity of NOTCH activation by NMHC, we compared our profiling data with the available microarray data (GSE42261) presenting the differential gene expression in AML upon NOTCH activation by DLL4, the canonical ligand of NOTCH receptor^14^,^15^. As a result, around 35% of genes regulated by NMHC were overlapped with DLL4-modulated genes (Fig. 3e). These intriguing findings suggest that the NMHC-mediated anti-AML effect is attributable, at least partially, to activation of the NOTCH signaling pathway.

### NMHC enhances the NOTCH signaling pathway

**Figure 4:**
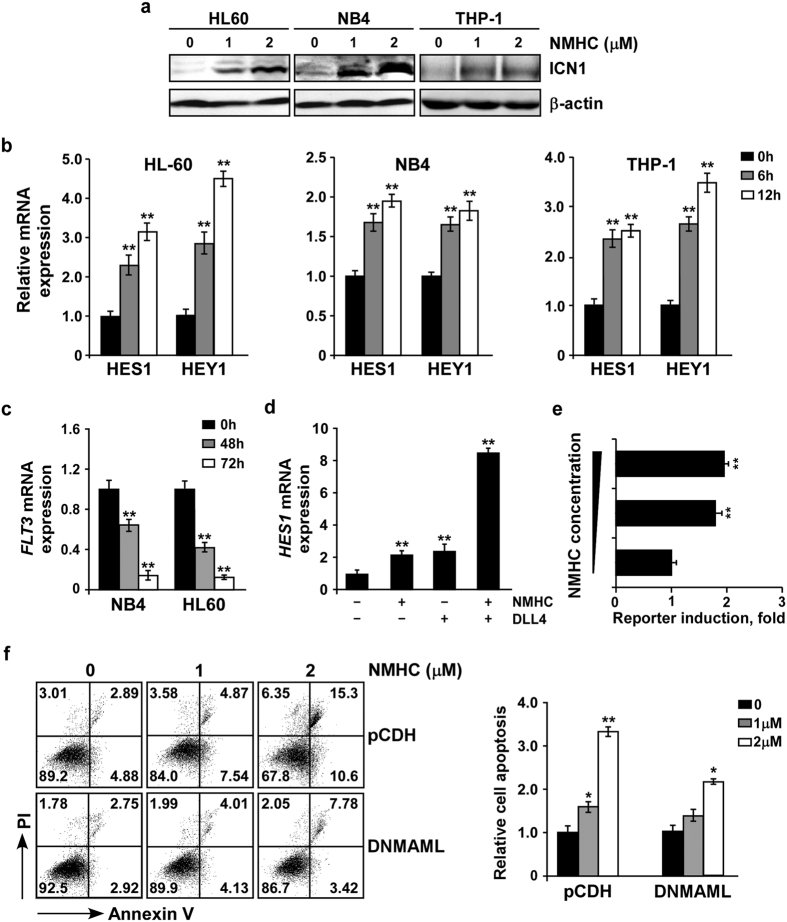
NMHC activated the NOTCH signaling pathway. (**a**) Effect of NMHC on ICN1 generation. HL-60, NB4 and THP-1 cells were treated with indicated doses of NMHC for 24 h. The amounts of ICN1 were detected by immunoblotting and normalized with the internal reference β-actin. (**b**) Effects of NMHC on the expression of NOTCH target genes. Cells were treated with 1μM NMHC for 6 h and 12 h. *HES1* or *HEY1* mRNAs were determined by RT-qPCR using *18s rRNA* as an internal control. Similarly, *FLT3* mRNA was analyzed upon 48 and 72 h NMHC treatments (**c**). (**d**) Effect of NMHC, DLL4 and NMHC combined with DLL4 on *HES1* expression in NB4. Cells were treated with DLL4 (10 μg/mL), or NMHC (1 μM), or combination of both for 24 h. *HES1* mRNA was then analyzed by RT-qPCR. For all quantitative PCR analysis, relative RNA abundance to *18s rRNA* is represented as a mean from triplicate wells ± SD. (**e**) NMHC activated the NOTCH luciferase reporter activity. Luciferase reporter genes harboring NOTCH responsive elements were transfected into 293T cells, NMHC was added 3 h post-transfection for additional 24 h. Relative luciferase reporter activity is represented as a mean from triplicate wells ± SD. (**f**) NOTCH inactivation renders AML cells less sensitive to NMHC. NB4 cells infected with lentivirus expressing pCDH or pCDH-DNMAML were selected with puromycin (2 μg/mL) for 48 h. Resulting puromycin-resistant cells were subjected to 24 h DMSO or NMHC treatments (1 and 2 μM), stained with Annexin V-APC/PI and analyzed by flow cytometry. Representative data are shown on the left and quantifications relative to untreated cells are on the right. Quantitation is derived from the mean ± SD from three independent experiments. *P*-values were derived from Student’s *t*-test (*P < 0.05, **P < 0.01; NMHC treatments vs DMSO treatments).

The microarray results prompted us to confirm whether NMHC caused a substantial NOTCH activation. To this end, we assessed the protein amounts of intracellular NOTCH1 (hereafter called ICN1), the activated form of NOTCH1 processed by γ-secretase^16^. HL-60, NB4 and THP-1 cells were cultured in the presence or absence of NMHC, then ICN1 were determined by western blotting. Whereas rare expression of ICN1 was observed in mock treated AML cells, administration of NMHC, in a dose dependent manner, caused a noticeable increase of ICN1 (Fig. 4a). Moreover, activation of the NOTCH signaling pathway in AML was confirmed by induction of the canonical NOTCH1 target genes *HES1* and *HEY1* (Fig. 4b and supplementary Fig. 1a). Similar to prior reports that HES1 suppresses *FLT3* transcription in AML^8^, we also detected down-regulation of *FLT3* upon NMHC treatments that is associated with the increased expression of *HES1* (Fig. 4c). Notably, combination of NMHC with recombinant human DLL4-Fc, a polypeptide encoding the well-established NOTCH1 ligand, executed a synergistic effect on NOTCH activation, evidenced by dramatic induction of *HES1* expression in the combined treatments (Fig. 4d). More importantly, DLL4 dramatically enhanced NMHC-mediated cytotoxicity (Supplementary Fig. 1b). We further evaluated the effect of NMHC on NOTCH activation in 293T cells expressing a luciferase reporter gene that bears NOTCH1 responsive *cis*-elements. Increasing concentrations of NMHC led to marked enhancement of luciferase reporter activity (Fig. 4e), providing compelling evidence that this compound is capable of stimulating NOTCH signaling.

To address whether NMHC-mediated growth inhibition was a consequence of NOTCH activation, we genetically inactivated endogenous NOTCH and assessed the effect of NMHC on cell proliferation. DNMAML that specifically turns off NOTCH^17^ was cloned in the pCDH lentiviral vector with GFP as a surrogate marker. NB4 or HL-60 cells were infected with lentivirus expressing vector alone or DNMAML, and DNMAML infected cells exhibited lower NOTCH1 target expression (Supplementary Fig. 1d). Whereas marked cell death was induced by NMHC in vector expressing NB4 cells, the presence of NMHC resulted in reduced apoptosis in DNMAML infected cells (Fig. 4f). Similarly, HL-60 cells expressing DNMAML were noticeably enriched upon NMHC treatments, while vector expressing cells remained unchanged (Supplementary Fig. 1e), suggesting that pre-inactivation of NOTCH renders AML cells more resistant to the chemical insults that promotes NOTCH activation. We thus conclude that NOTCH activation by NMHC partly accounts for its consequent cytotoxicity in AML.

### NMHC promotes NOTCH1 processing and ICN1 generation

**Figure 5:**
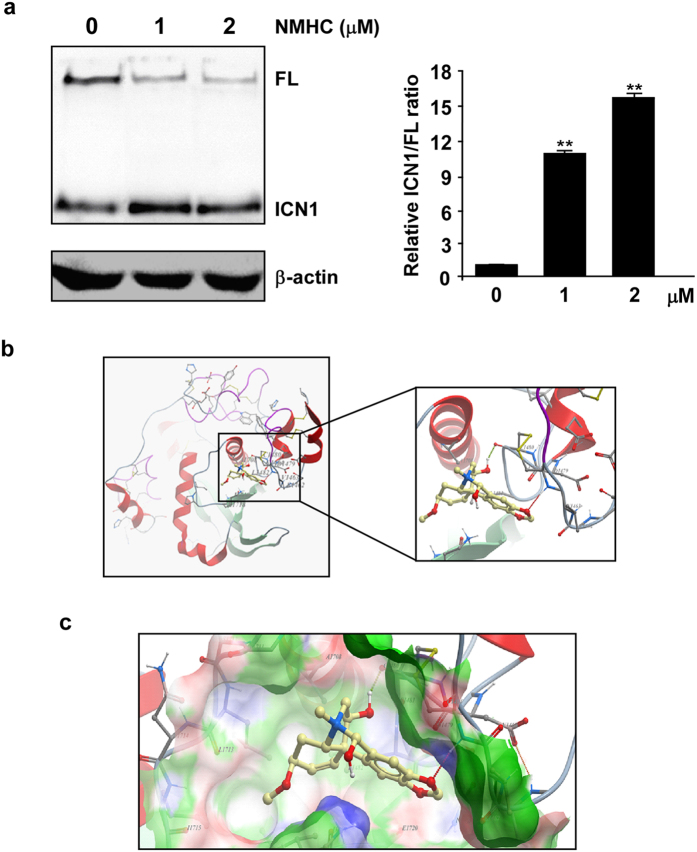
NMHC promoted NOTCH1 processing. (**a**) Effects of NMHC on the efficiency of ICN1 generation. HL-60 cells were treated with indicated doses of NMHC for 24 h, and the full length (FL) or cleaved NOTCH1 (ICN1) was detected by immunoblotting. Protein band intensity was quantified by ImageJ and ratios of ICN1/FL were calculated and plotted on the right. *P*-values were derived from Student’s *t*-test (*P < 0.05, **P < 0.01). (**b**,**c**) Cartoon representation of NMHC (yellow sticks) docked in the hydrophobic cavity of the NOTCH1 NRR (red and green). The X-ray crystal structures of the NRR domain was from PDB (code: 3ETO) and molecular modeling method was employed to investigate the interaction of NMHC with NRR. In b, the right panel is an amplification of the defined region in the box shown on the left.

Due to minimal changes of *NOTCH1* mRNA levels upon NMHC treatments (Supplementary Fig. 2), we hypothesized that the NMHC activation of NOTCH (Fig. 5) occurred at the post-transcriptional level. The NOTCH1 receptor processing was then evaluated by comparing protein abundance of full-length and cleaved intracellular portion. Notably, NMHC treatments of HL-60 diminished full-length NOTCH1 proteins in accompany with increased ICN1 production (Fig. 5a), suggesting that this compound facilitates proteolytic NOTCH1 cleavage and ICN1 release. We further employed the molecular modeling analysis based on the X-ray structure of NOTCH1 to simulate if NMHC incorporates into any NOTCH1 domains. Structures of extracellular NOTCH1 EGF repeats^18^,^19^,^20^, negative regulatory region (NRR) domain^21^,^22^,^23^ and intracellular ANK domain^24^,^25^ were studied by the Molsoft ICM method for compound incorporation. Among those, NMHC was revealed to dock perfectly in the hydrophobic pocket of NOTCH1 NRR (Fig. 5b,c). Immediately preceding the transmembrane segment, NRR maintains its closed and auto-inhibitory conformation that sterically occludes the metalloprotease cleavage^21^. The majority of known NOTCH1 activating mutations map to residues in the hydrophobic interior of NRR^21^,^26^. It is possible that incorporation of NMHC in NRR mimics the function of activating mutations identified in acute T-cell lymphoblastic leukemia (T-ALL), facilitating its proteolytic cleavage and the following release of active intracellular NOTCH1.

### *In vivo* effect of NMHC on a human AML xenograft model

**Figure 6:**
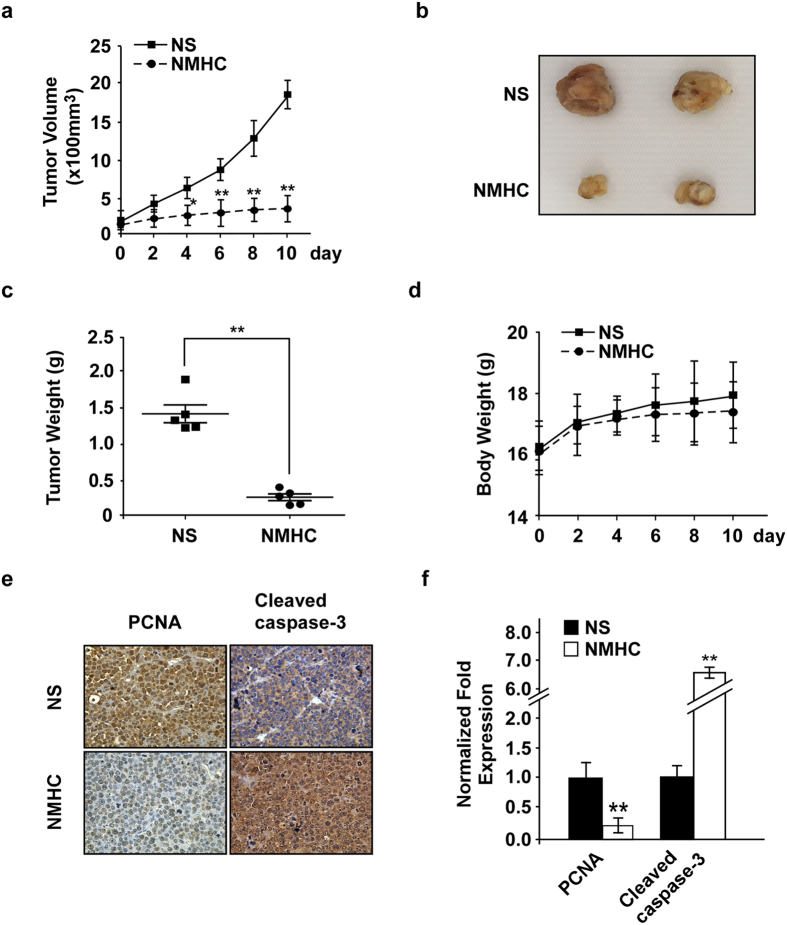
NMHC impeded tumor development in a human AML xenograft model. 5 × 10^6^ HL-60 cells were subcutaneously injected into female nude mice. When tumor volume reached 100 mm^3^, 40 mg/kg NMHC (n = 5) or NS (n = 5) was administrated once each day. (**a**) Tumor volumes were measured every other day post-injection and presented as square (untreated) or round (treated) symbols with mean values ± SD. (**b**) Representative tumor images were shown upon normal saline or NMHC treatments for 10 days. (**c**) Tumor weights were exhibited as square (untreated) or round (treated) symbols. The black bar in the middle represented the average tumor weight of each group. (**d**) Body weights were recorded post-treatments every other day and presented as mean values ± SD. Histochemical stains of cleaved Caspase-3 and PCNA in tumors from treated or untreated animals were shown in (**e**) and quantified by Ipwin32 software and presented as mean ± SD (**f**). *P*-values were derived from Student’s t-test (*P < 0.05, **P < 0.01; NMHC treatments versus control treatments).

To determine the anti-leukemia effect of NMHC *in vivo*, we established a human AML xenograft model. Five million HL-60 cells were injected subcutaneously at the right flank of female nude mice. Once the tumors grew up to 100 mm^3^, normal saline or NMHC (40 mg/kg) was intraperitoneally injected into tumor-bearing mice once per day. In comparison to the control, the cohorts injected with NMHC developed tumors with smaller volumes (Fig. 6a). At the day 10, the tumor volumes of control group reached an extent that mice begun sick, we then euthanized the animals and excised the tumors for weight measurements and histology analysis. Indeed tumor masses from the NMHC-treated individuals had reduced sizes (Fig. 6b) and lower tumor weights (Fig. 6c). Consistent with our prior studies^13^, injection of this small molecule did not affect body weights of tested animals (Fig. 6d), manifesting minimal toxicity from NMHC. To further manifest the effect of NMHC on tumor development, we assessed tumor cell proliferation and apoptosis *in vivo*, and detected decreased PCNA and enhanced cleaved Caspase-3 expression from the compound-treated tumors (Fig. 6e,f). Therefore, in consistence with cell-based experiments, NMHC exhibited strong anti-AML activity in mice.

## Discussion

Dysregulation of the NOTCH signaling pathway has emerged as a prevalent theme in leukemogenesis where its functional roles seem highly context-dependent. Whereas a major oncogene in T cell leukemia in humans and mice^26^, NOTCH plays a tumor suppressive role in acute myeloid leukemia^27^ and development of effective NOTCH agonist shows great promise to benefit AML patients. We and others previously reported that the chemical constituents of *Zephyranthes candida* (*Lindl.*) Herb displayed strong anti-neoplastic activities^13^,^28^. In this study, we determine NMHC, one of the key Amaryllidaceae alkaloids from *Zephyranthes candida*, possesses specific cytotoxicity in AML cells while spares B-ALL, T-ALL and CML cells. *In vivo* functional studies further demonstrate that NMHC treatment impedes AML development. Mechanistically, NMHC exerts its anti-leukemic activity through activation of the NOTCH signaling pathway. In aggregate, our findings establish NMHC as a potential novel NOTCH agonist, which may develop into therapeutic agents for AML treatments.

Several lines of evidence manifest that NOTCH plays a tumor suppressive role in AML^27^. NOTCH signaling inactivation leads to myeloproliferative disease in mouse models^6^. Multiple genes implicated in the NOTCH pathway are hypermethylated and silenced in IDH1/2 mutant AML^29^,^30^. NOTCH reactivation, mainly through enforced expression of activated NOTCH or application of polypeptides mimicking NOTCH ligands, successfully inhibit leukemogenesis *in vitro* and *in vivo*^5^. Given that the NOTCH pathway has emerged as a potential therapeutic target in AML, pharmacological NOTCH activation holds great promises. However, lentivirus or peptide-mediated NOTCH reactivation in AML patients remain limited in application owing to random viral insertion, peptide instability or low bioavailability^31^. In this regards, small molecules capable of activating NOTCH should be a better substitute.

Our data have provided compelling evidence showing the natural product NMHC has an intrinsic capacity to induce the NOTCH signaling pathway. The whole transcriptome profiling of HL-60 cells treated with NMHC reveals that genes involved in the NOTCH pathway are significantly enriched. Moreover, a large number of genes modulated by the NOTCH ligand DLL4 are also regulated by NMHC and the overlap of NOTCH signature genes provide a comprehensive evidence arguing that NMHC, mimicking NOTCH ligands, activates the NOTCH pathway. This effect is not merely limited in acute myeloid leukemia as NMHC consistently activates luciferase reporter gene harboring NOTCH responsive elements in 293T cells. Biochemical assays and structure-based molecular modeling further show that NMHC is capable of enhancing NOTCH receptor processing by binding to NRR domain, rendering the transmembrane receptor more susceptible to proteolytic cleavages. To provide strong support from the computer-based modeling, critical residues that mediates the NMHC interaction could be mutated and further examined whether they would abolish NOTCH activation. Alternatively, direct physical binding of NMHC and NOTCH1 NRR may be determined by co-crystallization of this compound with NRR or co-immunoprecipitation of biotin-labeled NMHC with AML cell extract. Regardless, all functional and mechanistic results provide strong evidence that NMHC has the capacity to enhance the NOTCH signaling pathway. As a small molecule compound, NMHC could be further developed as a unique NOTCH activator that is permeable and stable to replace the peptide-based NOTCH modulation.

The NMHC-mediated anti-AML effects were validated *in vitro* and *in vivo* and, more importantly, reversed by pre-inhibition of NOTCH activity, suggesting that NOTCH is a major target, if not all, of NMHC for its cytotoxic effects in AML. In this regard, we do not exclude the possibilities that NMHC affects alternative targets that mediates anti-neoplastic activity, including those gene signatures involved in AML identified from our transcriptome profiling. Our work nevertheless provides the first systematical analysis on NMHC-regulated gene expression in AML and raises a possibility of pharmacological activation of NOTCH. NMHC may be further developed for AML treatments alone or in combination with other agents. It may also offer therapeutic opportunities for other cancers where NOTCH serves a tumor suppressive role^32^, such as head and neck squamous cell carcinoma^33^,^34^ and skin cancer^35^.

## Materials and Methods

### Reagents

*N*-methylhemeanthidine chloride (NMHC) was isolated from the whole plants of *Z. candida* as previously described^10^. CCK8[WST-8 (2-(2-methoxy-4-nitrophenyl)-3- (4-nitrophenyl) -5-(2,4- disulfophenyl)-2H-tetrazolium, monosodium salt)] was purchased from Beijing Zoman Biotechnology Co, Ltd. Antibodies against cleaved NOTCH1 (V1744), cleaved Caspase-3, β-actin and p21 were purchased from Cell Signaling Technology; PARP, CyclinB1 were purchased from BD Biosciences. Anti-serum recognizing full-length NOTCH1 was kindly offered by Dr Warren Pear at the University of Pennsylvania. DLL4-Fc was obtained from Life Technologies.

### Cell culture

Human AML cell lines HL-60, NB4, THP-1, Kasumi-1, K562 and 293T cells were purchased from ATCC. KOPTK1 cells were obtained from Dr. Warren Pear (University of Pennsylvania). RS4;11 was provided by the Department of Hematology, Tongji Hospital, Wuhan, P.R.China. As stated in the provided information, cell lines were characterized by their karyotypes, images and specific gene expression. 293T and RS4;11 cells were cultured in DMEM (Hyclone) and α-MEM (Hyclone), respectively, all remaining cells in RPMI-1640 (Hyclone) supplemented with 10% FBS^36^. All cells were cultured at 37 °C in the presence of 5% CO_2_ and used for fewer than 6 months after resuscitation.

### Cytotoxicity assay

Cell viability was assessed via CCK8 assays according to the manufacturer’s instructions (Beijing Zoman Biotechnology Co, Ltd). 5000 cells in 200 μl were dispensed in each well of a 96-well plate prior to indicated NMHC treatments, then incubated in 20 μl CCK8 for 4 h at 37 °C. The absorbance of OD_450 nm_ was determined using a plate reader (Synergy HT).

### Cell cycle analysis

Cells were synchronized for 48 h in medium containing 0.2% FBS, cultured for 24 h in 10% FBS in the presence or absence of NMHC, washed in ice-cold PBS and fixed in 70% ethanol at 4 °C for 12 h, followed by PI (10 μg/mL) staining in the presence of 20 μg/mL RNase A. After 30 min incubation in the dark on ice, cell cycle distributions were analyzed by flow cytometry.

### Apoptosis assay

Apoptotic cell death was assessed as described^37^. After NMHC treatments, cells were collected and stained with Annexin V/PI in the dark at 4 °C for 30 minutes, then subjected to flow cytometry analysis.

### Western blot analysis

Cells were lysed in RIPA (Radio-Immunoprecipitation Assay) lysate buffer^38^, then protein concentration were quantified with BCA (bicinchoninic acid) protein assay reagents (ZOMANBIO). Equivalent lysate samples were loaded on SDS-PAGE and transferred to PVDF membranes (Bio-Rad). Immunoblotting was performed using indicated primary antibodies and HRP conjugated anti-mouse (or rabbit) IgG. Protein bands were detected by Supersignal West Pico Chemiluminescent Substrate (Thermo Scientific) and visualized using GeneGnome5 gel imaging and analysis systems (Synoptics Ltd.).

### Microarray analysis

AML cell line HL-60 was treated with or without 1 μM NMHC for 24 h, total RNA was extracted using the TRIzol (Invitrogen). cRNA was labeled and hybridized to the PrimeView^TM^ human gene expression array (Affymetrix). GSEA was performed using the Java GSEA implementation downloaded from www.broad.mit.edu/gsea/msigdb/ and gene sets used in the analysis were taken from the MSig Database of the Broad Institute^39^.

### Molecular docking

ICM-Pro 3.8.1 molecular docking software^40^ was employed to study the interaction of NMHC and NOTCH1, based on previously determined crystal structures of various NOTCH1 domains (PDB code: 3ETO, 3L95, 3I08, 2VJ3, 4CUE, 4CUF, 4CUD, 4CUE, 4CUF, 4D0E, 4D0F, 2HE0, 2F8Y, 1YYH). In the docking calculation, potential energy maps of each domain were obtained using default parameters. Conformational sampling was based on the Monte Carlo procedure to identify the binding site with the lowest-energy and most favorable orientation of NMHC.

### RNA extraction and quantitative real-time PCR

Quantitative PCR was performed as previously described^36^. Briefly total RNA extracted with TRIzol was reverse transcribed using random primers and the Revert Aid First strand cDNA synthesis kit (Thermo Scientific) according to the manufacturer’s instruction. Real-time PCR was conducted using FAST SYBR Green Master Mix (Bio-Rad) on CFX Connect Real-Time PCR System (Bio-Rad) and 18s rRNA was used as a control. Primers were shown as following. *18s rRNA* forward primer: 5′-GCGCCGCTAGAGGTGAAAT-3′ and reverse primer: 5′-GGCGGGTCA TGGGAATAAC-3′; Human *HES1* forward primer: 5′-TCAACACGACACCGGAT AAA-3′ and reverse primer: 5′-TCAGCTGGCTCAAGACTTTCA-3′; Human *HEY1* forward primer: 5′-AGCAGGTAATGGAGCAAGGA-3′ and reverse primer: 5′-CGCGTCAAAGTAACCTTTCC-3′; Human *FLT3* forward primer: 5′-TGCCGC TGCTCGTTGTT-3′ and reverse primer: 5′-AGGTCTTCCGGGGA TTCTGAT-3′. All PCR reactions were performed in triplicate and relative expression of the mRNA was determined using 2^−ΔΔCt^ method.

### Lentiviral transduction

pCDH vector or pCDH encoding dominant negative MAML (DNMAML) was co-transfected into 293T with helper plasmids (pMD2.G and psPAX2)^41^,^42^. Lentiviral supernatant was collected to infect AML cells, supplemented with 6 μg/mL polybrene and centrifuged at a speed of 2500 rpm for 1 h at room temperature. After 48 h incubation, GFP^+^ cells were detected by flow cytometry and/or purified by puromycin (2μg/mL) for additional 48 h.

### Luciferase reporter gene assays

250 ng of firefly luciferase reporter (pGL3-promoter) constructs with pTα enhancers^36^ was co-transfected with 50 ng of Renilla luciferase constructs into 293T cells using Lipofectamine 2000 (Invitrogen). Luciferase reporter activities were measured 24 h later using the Dual Luciferase Reporter Assay (Promega). Firefly luciferase activities were normalized with Renilla luciferase control values. Relative activity from the empty vector lysate was set arbitrarily to a value of 1.

### AML xenograft models

Four to six week female BALB/C nude mouse (Beijing HFK Bioscience Co., Ltd) were subcutaneously injected at the right flank with 5 × 10^6 ^HL-60 cells suspended in 200 μl PBS containing 50% Matrigel^TM^ Matrix (BD BioSciences). When the tumors reached the volume of 100 mm^3^, animals were randomly divided into two groups. NMHC-treated mice were injected intraperitoneally with a dose of 40 mg/kg every day, while the control cohort were injected equal volume of solvent natural saline (NS)^13^. To avoid DMSO-mediated cytotoxicity *in vivo*, we resuspended 60 mg NMHC with 15 μl DMSO and further dissolved to desired concentrations in natural saline prior to animal injection. Tumor volumes were calculated as the formula: a × b^2^/2, where *a* represents the largest diameter and *b* the smallest diameter. Animals were maintained in specific pathogen-free conditions within the animal care facility at the Experimental Animal Center of Huazhong University of Science and Technology. All the experimental protocols were approved by the Animal Experimentations Ethics Committee of Tongji Medical College and conducted in accordance with the Guidelines for the Care and Use of Laboratory Animals of Tongji Medical College of Huazhong University of Science and Technology.

### Immunohistochemistry

The immunohistological analysis was performed using the Histostain^TM^-Plus Kit (Invitrogen) as previously described^37^. Tumor sections were stained with the primary antibodies against PCNA and cleaved Caspase-3 overnight at 4 °C, then incubated with the appropriate biotinylated secondary antibodies for 30 min and HRP-linked streptavidin agents for 15 min at room temperature. Stains were visualized by the DAB substrate kit (Vector Labs) and quantified by the Ipwin32 software.

### Statistical analyses

Statistics was analyzed by the student *t* test. *P* < 0.05 were considered statistically significant.

## Additional Information

**How to cite this article**: Ye, Q. *et al.* Small molecule activation of NOTCH signaling inhibits acute myeloid leukemia. *Sci. Rep.* **6**, 26510; doi: 10.1038/srep26510 (2016).

## Supplementary Material

Supplementary Information
