# Supplementary material for: Small molecule activation of NOTCH signaling inhibits acute myeloid leukemia
Source: Sci Rep. 2016 May 23;6:26510. doi: 10.1038/srep26510 (PMC4876435; doi:10.1038/srep26510)
Supplement: Supplementary Information [file srep26510-s1.pdf]

Small molecule activation of NOTCH signaling inhibits acute myeloid leukemia

Qi Ye [1,2,†], Jue Jiang[1,†], Guanqun Zhan[1,†], Wanyao Yan[1], Liang Huang[3], Yufeng Hu[1], Hexiu Su[1], Qingyi Tong[1], Ming Yue[4], Hua Li[1], Guangmin Yao[1,*], Yonghui Zhang[1,*], Hudan Liu[5,*]

[1]Hubei Key Laboratory of Natural Medicinal Chemistry and Resource Evaluation, School of Pharmacy, Tongji Medical College, Huazhong University of Science and Technology, Wuhan, 430030, China; [2]Department of Pharmacy, Wuhan Children's Hospital, Wuhan, 430014, P. R. China; [3]Department of Hematology, Tongji Hospital, Wuhan, 430030, China; [4]School of Basic Medicine, Tongji Medical College, Huazhong University of Science and Technology, Wuhan, 430030, China; [5]Medical Research Institute, Wuhan University, Wuhan, 430071, China. [†]These authors contributed equally to this work. Correspondence and requests for materials should be addressed to G.Y. (email: gyap@hust.edu.cn) or Y.Z. (email: zhangyh@mails.tjmu.edu.cn) or H.L. (email: hudanliu@whu.edu.cn)

Supplementary Figure 1

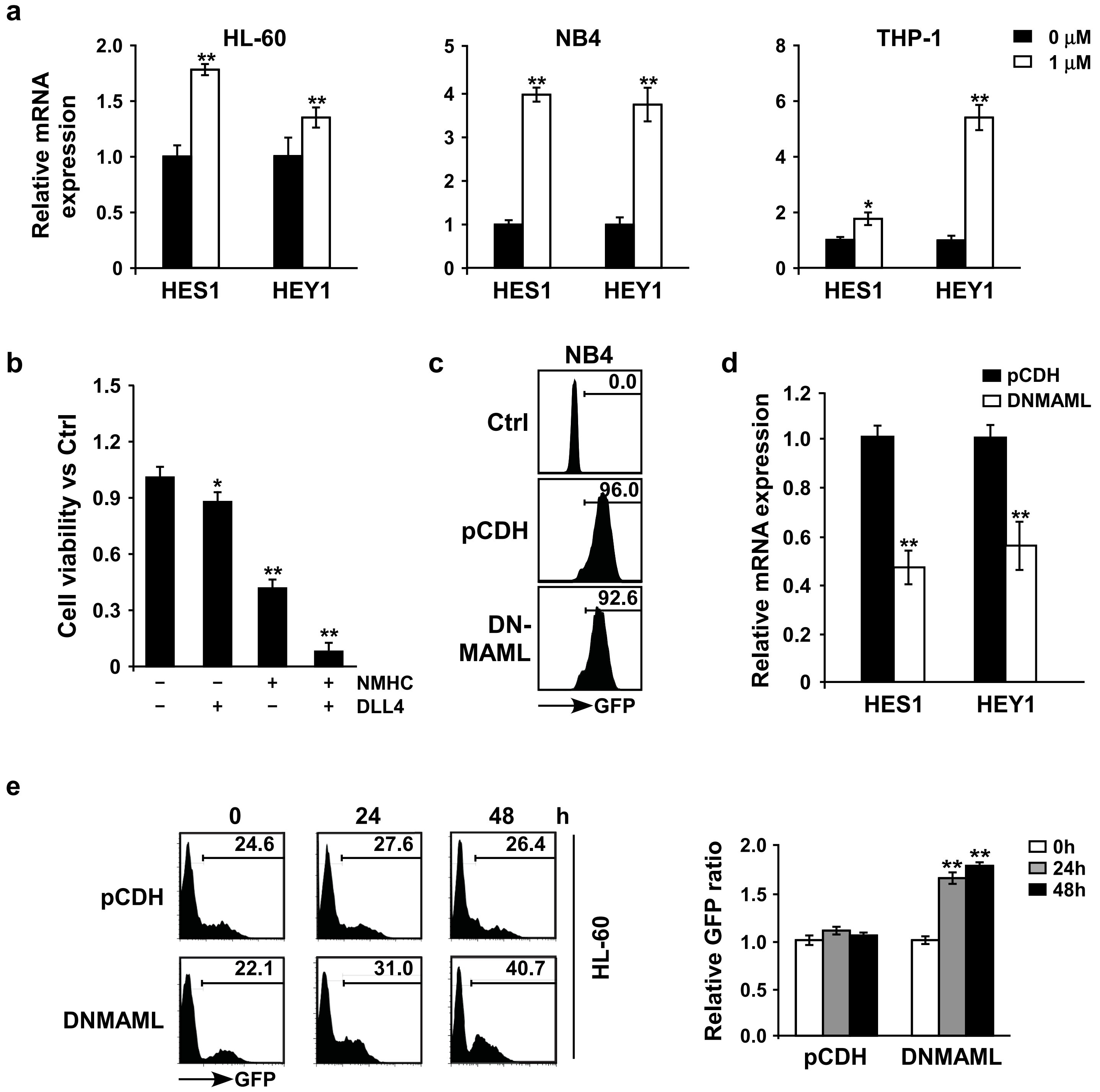

Figure S1. NMHC promotes NOTCH activity and NOTCH activation-mediated AML inhibition. (a) Effects of extended NMHC exposure on NOTCH target gene expression. Cells were treated with 1μM NMHC for 48 h. *HES1* and *HEY1* mRNAs were determined by RT-qPCR as described in Fig. 4. **(b)** Synergistic cytotoxic effect of NMHC in combination with DLL4. NB4 cells were treated with DLL4 (10 μg/mL), NMHC (1 μM) or both for 24 h. Cell viability was detected by CCK8 and presented as a ratio relative to untreated samples. Data shown is the mean ± SD from triplicate wells**. (c)** NB4 cells infected with pCDH and pCDH-DNMAML were selected by puromycin. As pCDH vector has GFP as a surrogate marker, we ensured cell purity by detecting GFP^+ percentages. **(d)** NOTCH inactivation downregulated target gene expression. Infected cells from **(c)** were harvested for RT-qPCR and NOTCH1 targets, such as *HES1* and *HEY1*, were analyzed and presented as shown. Data shown is the mean ± SD from triplicate wells. **(e)** NMHC enriched AML cells with NOTCH inactivation. HL-60 cells infected with pCDH or pCDH-DNMAML were subjected to NMHC (1 μM) treatments 2 day post-infection. GFP^+ distributions were analyzed by flow cytometry at the indicated time points (left). GFP ratio relative to untreated cells was presented (right). Data shown is the mean ± SD from three independent experiments. Above all, *p*-values were derived from Student's *t*-test (**p* < 0.05, ***p* < 0.01).

Supplementary Figure 2

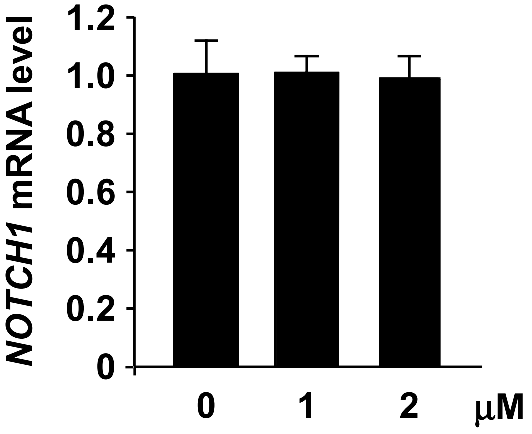

Figure S2. *NOTCH1* mRNA levels in AML cells are unaffected by NMHC. HL-60 cells were subjected to 1 or 2 M NMHC treatments for 24 h, then harvested for reverse transcription. *NOTCH1* mRNA amounts were determined by qPCR, normalized with *18s rRNA*. qPCR primers used to detect *NOTCH1* are CCGCAGTTGTGCTCCTGAA and ACCTTGGCGGTCTCGTAGCT.
